# 

**Published:** 1898-03

**Authors:** 


**ESTABLISHED 1855.** **SUBSCRIPTION, $2.00.**

### ATLANTA MEDICAL AND SURGICAL JOURNAL.

VOLUME XV NEW SERIES. ATLANTA, GA., MARCH, 1898. No. 1.

